# Phylogeny and Species Delimitation of Chinese *Medicago* (Leguminosae) and Its Relatives Based on Molecular and Morphological Evidence

**DOI:** 10.3389/fpls.2020.619799

**Published:** 2021-01-13

**Authors:** Jinyuan Chen, Guili Wu, Nawal Shrestha, Shuang Wu, Wei Guo, Mou Yin, Ao Li, Jianquan Liu, Guangpeng Ren

**Affiliations:** ^1^State Key Laboratory of Grassland Agro-Ecosystems, School of Life Sciences, Institute of Innovation Ecology, Lanzhou University, Lanzhou, China; ^2^Key Laboratory of Bio-Resource and Eco-Environment of Ministry of Education, College of Life Sciences, Sichuan University, Chengdu, China

**Keywords:** DNA barcodes, integrative approach, *Medicago*, *Melilotus*, phylogeny, *Trigonella*

## Abstract

*Medicago* and its relatives, *Trigonella* and *Melilotus* comprise the most important forage resources globally. The alfalfa selected from the wild relatives has been cultivated worldwide as the forage queen. In the Flora of China, 15 *Medicago*, eight *Trigonella*, and four *Melilotus* species are recorded, of which six *Medicago* and two *Trigonella* species are introduced. Although several studies have been conducted to investigate the phylogenetic relationship within the three genera, many Chinese naturally distributed or endemic species are not included in those studies. Therefore, the taxonomic identity and phylogenetic relationship of these species remains unclear. In this study, we collected samples representing 18 out of 19 Chinese naturally distributed species of these three genera and three introduced *Medicago* species, and applied an integrative approach by combining evidences from population-based morphological clusters and molecular data to investigate species boundaries. A total of 186 individuals selected from 156 populations and 454 individuals from 124 populations were collected for genetic and morphological analyses, respectively. We sequenced three commonly used DNA barcodes (*trnH-psbA*, *trnK-matK*, and ITS) and one nuclear marker (*GA3ox1*) for phylogenetic analyses. We found that 16 out of 21 species could be well delimited based on phylogenetic analyses and morphological clusters. Two *Trigonella* species may be merged as one species or treated as two subspecies, and *Medicago falcata* should be treated as a subspecies of the *M. sativa* complex. We further found that major incongruences between the chloroplast and nuclear trees mainly occurred among the deep diverging lineages, which may be resulted from hybridization, incomplete lineage sorting and/or sampling errors. Further studies involving a finer sampling of species associated with large scale genomic data should be employed to better understand the species delimitation of these three genera.

## Introduction

Species is the basic unit of biodiversity (Mayr, 1982; Claridge et al., 1997; Coyne and Orr, 2004) and almost all the biological researches regard one or more species as their study subjects (Guggisberg et al., 2006; Zheng et al., 2014; Pastore et al., 2019). Although there are at least 30 recognized concepts on species (Wilkins, 2009), most of them are incomplete as they only reflect one or two characters of species. A clear-cut consensus on species has not been reached yet (Zhou and Yang, 2011). A common assumption in several species concepts is that the species represent distinct evolutionary units with limited gene flow to other species, and they exhibit concordance among different character sets (Avise, 1990). Recent theory has suggested that all species are in the way of becoming divided, frequently species beginning the next divide when it does not complete the current differentiation (Queiroz, 2007; Liu, 2015). Gene flow between related species also occurs more frequently than previously thought (Wu and Ting, 2004; Liu, 2016), which leads to ambiguous species boundaries, especially between closely related species. Moreover, the widespread occurrence of incomplete lineage sorting and interspecific introgression among related lineages (i.e., incomplete reproductive isolation) makes species delimitation more challenging. It may, therefore, be insufficient to use only one species concept. It may rather be more appropriate to use integrative species concepts (Liu, 2016; Hong, 2020), which reconciles different sources of data and meets multiple criteria of the diverse species concepts. Recent studies based on statistical analysis of morphological variation within and between species, combined with molecular evidence, mostly DNA barcodes (e.g., *psbA-trnH*, *trnL-F*, and ITS) at the population level, have provided strong power and new insights in delimiting species boundaries in many taxa, such as *Orinus* (Su et al., 2015), *Orychophargmus* (Hu et al., 2015), *Ostrya* (Lu et al., 2016), and *Stachyuraceae* (Su et al., 2020). Here, we apply such an integrative approach to facilitate species delimitation in *Medicago* L. and its two related genera.

*Medicago* and its relatives, *Trigonella* L. and *Melilotus* Miller, belonging to the subtribe Trigonellinae (Leguminosae), are three genera that are widely distributed throughout the Eurasia and in the North Africa and Oceania, with the Mediterranean regions as their diversity center. The three genera are closely related and the latter two genera were included in *Medicago* sensu lato by some authors (Small and Jomphe, 1989; Bena, 2001). However, here, we follow Flora of China and treat them as three tentative genera (Xu et al., 2010). The three genera are famous for fine pasture especially the alfalfa (*Medicago sativa* L.), which is known as “the queen of forage crops” (Small, 2010) and the biological model species, *Medicago truncatula*. Some species of these genera are also used for food and medicines. For example, fenugreek (*Trigonella foenum-graecum* L.) and alfalfa are widely used in the treatment of diabetes, menstrual cramps and high cholesterol (Malinow et al., 1978, 1980; Hong et al., 2009; Bora and Sharma, 2011; Seida et al., 2015; Sadeghi et al., 2016; Wani and Kumar, 2018). Generic circumscription has long been problematic in the tribe Trifolieae subtribe Trigonellinae (Bena, 2001), especially between *Medicago* and *Trigonella* concerning the “medicagoid” species, which has long been considered as belonging to the genus *Trigonella* due to the strong similarities in the appearance of their fruit. However, Baum (1968) named 23 species of *Trigonella* as “medicagoid” species due to similarities in flower and seed structure with those of *Medicago*. Later, Small (1987) proposed to transfer these 23 “medicagoid” species to *Medicago* because of the shared explosive tripping pollination mechanism. This is partly supported by the phylogeny based on ITS and ETS in which 10 “medicagoid” species are grouped with the *Medicago* clade rather than the *Trigonella* clade (Bena, 2001). In addition, phylogenetic relationships within and between these three genera are also obscure and unresolved, and molecular evidence based on DNA barcodes often contradict the sectional delimitation based on morphology (e.g., within *Trigonella* see Dangi et al., 2016; within *Medicago* see Hu et al., 2014, and within *Melilotus* see Di et al., 2015). Phylogenetic analyses using DNA barcodes or whole genome resequencing data for *Medicago* at species level recovered significant incongruent species relationships among recently diverged taxa between gene phylogenies (Bena, 2001; Maureira-Butler and Pfeil, 2008; Steele et al., 2010; Yoder et al., 2013; Sousas et al., 2016), suggesting that incomplete lineage sorting and/or interspecific introgression are widespread within the genus. However, few studies have tested the competing hypotheses of species delimitation in *Medicago* and *Trigonella* using DNA barcodes at the population level.

DNA barcodes, such as *trnL-F*, *rbcL*, *matK*, *trnK-matK*, *psbA-trnH*, and ITS, are developed to distinguish between both closely and distantly related species of different genera and families (Hebert et al., 2003; Cowan et al., 2006; CBOL Plant Working Group et al., 2009). However, the discrimination power of these barcodes varies greatly depending on the studied groups (e.g., Hu et al., 2015; Lu et al., 2016). Previous study suggested that *trnL-F*, *rbcL*, and *matK* seemed not sufficient to delimit the species of *Melilotus* (Di et al., 2015), while at most scenarios, ITS was found to be effective in discriminating the closely related species with the relatively recent divergences (Li et al., 2011). Furthermore, *trnK-matK* and one nuclear gene marker (*GA3ox1*) were found to be informative for phylogenetic resolution at species level within *Medicago* (Steele et al., 2010; Hu et al., 2014). Therefore, in this study, we used those DNA barcodes (ITS, *trnK-matK* and *psbA-trnH*) and the nuclear DNA (nrDNA) marker (*GA3ox1*), and combined morphological variation with molecular evidence to examine phylogenetic relationships and species delimitation of *Medicago*, *Trigonella*, and *Melilotus*, using species from China as a case study.

According to the Flora of China^1^, 15 *Medicago* species (including one hybrid species *Medicago × varia*, and two “medicogoid” species, *Trigonella monantha* and *Trigonella orthoceras*, which are already treated as *Medicago monantha* and *Medicago orthoceras*), eight *Trigonella* species and four *Melilotus* species are recorded (Xu et al., 2010). Among them, eight (i.e., *Medicago arborea*, *M. sativa*, *Medicago × varia*, *Medicago praecox*, *Medicago arabica*, *Medicago polymorpha*, *Trigonella caerulea*, and *T. foenum-graecum*) are introduced and widely cultivated in China. Some of them (e.g., *M. sativa*, *Medicago × varia*, and *M. polymorpha*) have been escaped to roadsides, fields and stream banks and adapted to variable environments. The remaining species are naturally distributed in China and adjacent countries, and only *Medicago archiducis-nicolai* is endemic in China. This endemic species together with other three Chinese species (i.e., *Medicago ruthenica*, *Medicago platycarpos*, and *Medicago edgeworthii*) belong to the sect. *Platycarpae*. Phylogenetic analysis based on three DNA markers (ITS, *trnK-matK* and *GA3ox1*) indicated that *M. archiducis-nicolai* is clustered with *M. ruthenica* and *M. platycarpos*, but *M. edgeworthii* is clearly not nested within the sect. *Platycarpae* clade (Hu et al., 2014). In addition, the phylogenetic relationships of the other two “medicogoid” species (*Trigonella arcuata* and *Trigonella cancellata*) and other four *Trigonella* (*Trigonella cachemiriana*, *Trigonella pamirica*, *Trigonella emodi*, and *Trigonella fimbriata*) remains unclear. To examine the delimitation and relationships among these Chinese species, we sequenced three DNA barcode regions (ITS, *trnK-matK* and *psbA-trnH*) and one nuclear marker (*GA3ox1*), and remeasured 19 morphological traits at the population level. Specifically, we aimed to address the following questions: (1) Which marker is the most effective for phylogenetic resolution? (2) Do the two “medicogoid” species (*T. arcuata* and *T. cancellata*) also cluster with the *Medicago* clade? (3) How many species should be recognized in the three genera in China based on the integrated results of molecular and morphological variation? (4) Are the phylogenies reconstructed by nrDNA regions and chloroplast DNA (cpDNA) fragments congruent?

## Materials and Methods

### Plant Sampling

**FIGURE 1:**
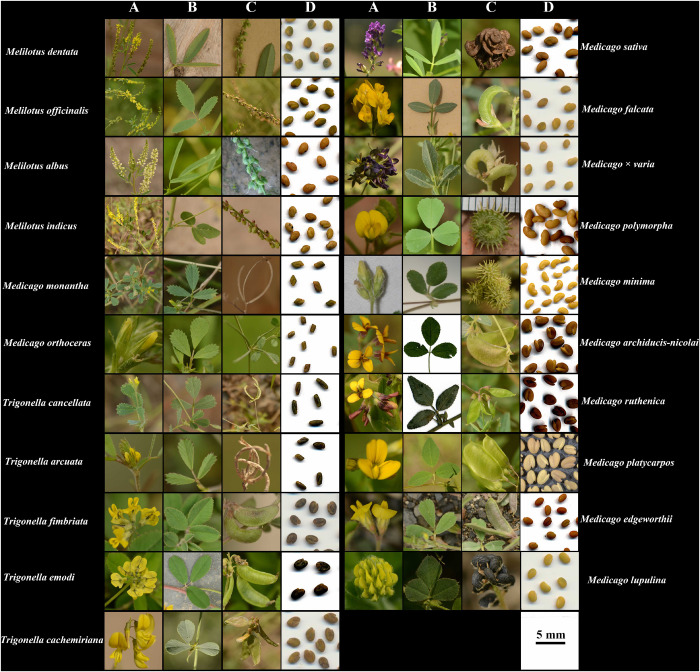
Photos of flowers **(A)**, leaves **(B)**, pods **(C)**, and seeds **(D)** of the 21 species analyzed in this study. All photos were taken by JC.

For the naturally distributed species of the three genera in China, we sampled 18 out of 19 species (94.7%) but failed to find the species *T. pamirica*. In addition, we also collected three introduced species (i.e., *M. sativa*, *M. polymorpha*, and *Medicago × varia*) because of their significant values as legume forage and included the well-known model species *M. truncatula* in our analyses, resulting in a total number of 22 species (Figure 1). For the species that are widely distributed in China, we collected 3–27 populations for each species (Supplementary Table 1) in the field according to the records in National Specimen Information Infrastructure^2^. For *M. platycarpos*, *T. arcuata*, *T. emodi*, *T. fimbriata*, and *T. cachemiriana* that only occur (but also in other countries) in the western or southwestern borders of China, we could only collect one or two populations for each species (Supplementary Table 1). We also obtained five samples of *T. emodi* and three samples of *T. fimbriata* from the specimens in Kunming Institute of Botany Herbarium (KUN). We followed standard protocol and collected the individual samples that were at least 20 meters apart in naturally distributed populations. In total, we collected fresh leaves and specimens for all the 21 species from 156 localities in China. We used silica gel to dry the fresh materials, and deposited all voucher specimens in Lanzhou University. The elevation, latitude, and longitude of locations were recorded using the mobile phone application “GPSkit v.2.3.9.” We selected 1–6 individuals from each of the 156 populations for molecular analysis, which resulted in a total of 186 individuals. The detailed information for the sampling localities and individuals used for molecular analysis was listed in Supplementary Table 1.

### DNA Isolation, Amplification and Sequencing

The whole genomic DNA of all samples was isolated from nearly 30 mg dried leaves according to the TIANGEN plant genomic DNA Kit (TIANGEN, Beijing, China) following the manufacturer’s instructions. We used previously published primers (Sang et al., 1997; Bena, 2001; Steele et al., 2010) of two cpDNA fragments (*psbA-trnH* and *trnK-matK*) and two nrDNA regions (*GA3ox1* and ITS) to amplify and sequence 166–186 individuals of the 21 species.

We performed the PCR amplification in 25 μL volume containing 1 μL of plant DNA with 50–150 ng/μL, 12.5 μL 2 × TSINGKE^®^ Master Mix (TIANGEN, Beijing, China) containing dNTPs, rTaq polymerase and 10 × PCR buffer, 10 μM of two primers and ddH_2_O added to 25 μL. For *trnK-matK* with nucleotide length of more than 2500 bp, multiple primer pairs were used for PCR amplification and sequencing (Supplementary Table 2). The PCR cycling parameters were coded for an initial denaturation step at 94°C for 5 min, followed by 35 cycles of 45 s at 94°C, 1 min 30 s at Tm (annealing temperature for each primer pair was shown in Supplementary Table 2), 45 s at 72°C, and the final extension step for 10 min at 72°C. The PCR products were examined through the agarose gel electrophoresis. The sequencing was carried out by TSINGKE Biotech (Xi’an, China) using the forward and reverse primer pairs. All sequences were submitted to GenBank (accessions MW241635-MW242342).

### Multiple Sequence Alignment and Phylogenetic Analyses

Multiple sequence alignment was performed using MEGA v.7.0 (Kumar et al., 2016) and revised manually. The sequenced fragments were joined in SeqMan v.7.1.0 (Burland, 2000). The two cpDNA and two nrDNA markers were further concatenated, respectively for phylogenetic analysis. Haplotypes, insertions/deletions (indels) and single nucleotides polymorphisms (SNPs) were identified using DnaSP v.5.0 (Librado and Rozas, 2009). Phylogenetic analyses for each marker and the concatenated cpDNA and nrDNA fragments were conducted by maximum likelihood (ML) using RAxML v.1.0.8 (Lemoine et al., 2018) and Bayesian inference (BI) using MrBayes v.3.2.7 (Ronquist and Huelsenbeck, 2003). The results of phylogenetic analyses for each marker were used to evaluate their relative discrimination power. The GTR + G + I model of sequence evolution was selected on the basis of Akaike information criterion (AIC) for all DNA markers and the two concatenated datasets as estimated by jModelTest v.2.1.7 (Guindon and Gascuel, 2003). Analyses of the data sets for ML were performed using the GTR + G + I model and 1,000 bootstrap replicates to evaluate the reliability of each internal branch. For BI analyses, we used the same model of sequence evolution selected by jModelTest. We repeated each analysis three times (i.e., each of the four markers and the two concatenated datasets) and each analysis consisted two parallel runs and four chains of 2,000,000 generations, sampling every 500 generations and 500,000 generations as burn-in. The convergence was determined by examining trace plots of the log-likelihood values for each parameter in Tracer 1.5. Sequences for the four markers of the model species *M. truncatula* and four species of genus *Trifolium* (*Trifolium albopurpureum* Torr. & A. Gray, *Trifolium incarnatum* L., *Trifolium semipilosum* Fresen. and *Trifolium subterraneum* L.) that were selected as outgroup (Supplementary Table 3) were downloaded from GenBank for the phylogenetic analyses. The congruence of the two trees reconstructed based on the two concatenated datasets was estimated following the methods described by Avino et al. (2019) using the R package “dendextend” (*tanglegram* function; Galili, 2015).

### Morphological Measurements of the Variable Traits

In order to test for consistently morphological differences for all the 21 species and *M. truncatula*, based on the major morphological traits used to establish species in the Flora of China (Xu et al., 2010) and by Small (1987), we re-examined seven characters for the vegetative organs (mature leaf length, mature leaf width, petiole length, serrate number of leaf margin, stipule length, serrate number of stipule margin, stipule shape), and 12 characters for the reproductive organs (pedicel length, inflorescence length, number of flowers in inflorescence, pod length, pod width, pod shape, number of seeds in pod, spiral number of pod, presence/absence of hair covering pod, flower color, flower length, carine length greater than wing length or not). To cover the morphological variation among and within populations, for the species that sampled multiple populations, we selected 3–12 individuals from each population for morphological measurements (Supplementary Table 4). For species that sampled only one population, 5–12 individuals for each species were measured. In total, 454 individuals representing 124 populations were used for principal component analysis (PCA) to detect morphological clusters. For each quantitative character, we measured three times per specimen and used the mean of the real values. For qualitative characters (i.e., stipule shape, pod shape, presence/absence of hair covering pod, flower color, and carine length greater than wing length or not), we used numbers (e.g., 0, 1, 2, 3) to represent the presence, absence, difference shapes or different colors of flower (Supplementary Table 4).

We first did PCA using all the measured characters of 22 species for a preliminary analysis and found that species belonging to *Medicago*/*Trigonella* and *Melilotus* formed two clusters, respectively, and none of the 22 species could be delimited (Supplementary Figure 1). This may be because our sampling represented multiple sections of three different genera, the levels of morphological variation at higher taxonomic level (i.e., between genera and sections) would be very high, which would cover up the variation at lower taxonomic level (i.e., among closely related species), leading to the failure of delimitation of closely related species. Therefore, it was not appropriate to perform the statistical analyses based on all the 22 species together. In order to establish morphological clusters based on statistical analyses of morphological characters at the population level, we assigned the 22 species into five groups on the basis of the five clades inferred by the cpDNA phylogeny (see section “Results”) and retained the characters that were shown variation among and within species of each clade. Finally, PCA was performed for each clade with the *princomp* function in “ggbiplot” package Vu (2011) in R to identify morphological clusters.

## Results

### Sequence Characteristics

In total, 708 new DNA sequences were generated in this study, including 167 *psbA-trnH* (497 bp, 10.2% missing data), 186 *trnK-matK* (2565 bp, no missing data), 177 *GA3ox1* (1311 bp, 4.8% missing data), and 178 ITS (622 bp, 4.3% missing data). The length of the four aligned markers ranged from 497 bp for *psbA-trnH* to 2565 bp for *trnK-matK* (Table 1). Among the four markers, ITS had the highest percentage of variable sites (SNPs; 20.57%) and nucleotide diversity (Pi, 0.05079) and *trnK-matK* the lowest (8.34% and 0.02002; Table 1). A total of 24 and 39 haplotypes were identified in the concatenated cpDNA and nrDNA datasets (Supplementary Table 5), respectively. The nrDNA dataset had the highest haplotype diversity (0.956).

**TABLE 1:** The characteristics, nucleotide diversity and haplotype diversity of the four DNA markers.

	Sample Size	Sequence length/bp	No. SNPs	% SNP	Nucleotide diversity (Pi)	Number of haplotypes	Haplotype diversity (Hd)
*trnK-matK*	186	2565	214	8.34%	0.02002	22	0.922
*psbA-trnH*	167	497	69	13.14%	0.03751	16	0.907
cpDNA	186	3062	283	9.24%	0.02006	24	0.930
*GA3ox1*	177	1311	118	9%	0.04635	30	0.950
ITS	178	622	128	20.57%	0.05079	27	0.933
nrDNA	178	1937	246	12.7%	0.04803	39	0.956

### Phylogenetic Analyses of DNA Datasets

**FIGURE 2:**
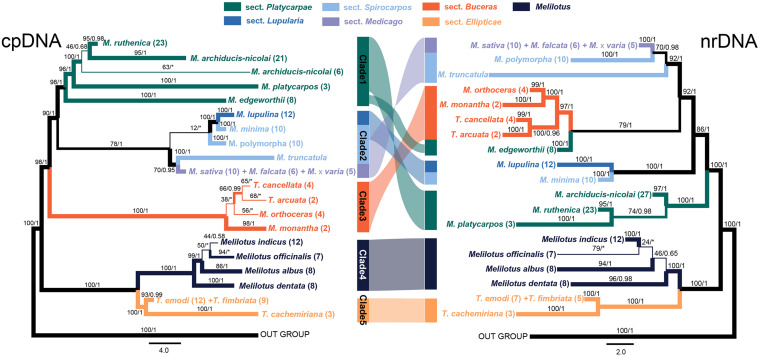
Phylogenetic trees of the three genera, *Medicago*, *Trigonella*, and *Melilotus* reconstructed based on two cpDNA barcodes (left) and two nuclear markers (right). Colors represent *Melilotus* and different sections of *Medicago* and *Trigonella*. Maximum likelihood (ML) bootstrap values and Bayesian posterior probabilities are indicated. The thickest branches indicate high support from the ML and BI analyses (bootstrap values ≥80 and posterior probabilities ≥0.95), thinner branches indicate high support from one of the two analyses, and the thinnest branches indicate low support from both analyses. The congruence and incongruence between the two trees are represented by color-shaded connections. The two inverted connections indicate significantly incongruence interspecific-relationships within each clade.

The ML and BI tree of each data set recovered congruent topologies except for some low supported groups, but discrepancies were obtained between the two markers (i.e., cpDNA and nrDNA), both at the independent and concatenated marker levels (Figure 2 and Supplementary Figure 2). The phylogenies reconstructed based on the four markers recovered different delimitating efficiency and phylogenetic relationships. The *GA3ox1* with the longest length of sequence (Table 1) had the strongest power in species delimitation, as indicated by higher supported values at nodes compared to the phylogenetic trees based on other three markers (Supplementary Figure 2). The nuclear ITS and cpDNA *trnK-matK* also showed considerable power in delimiting most of the 21 species, except for some closely related species. The cpDNA *psbA-trnH* showed the lowest discrimination power. In the concatenated cpDNA phylogenetic tree, five monophyletic clades were inferred (Figure 2). Clade 1 included four species belonging to *Medicago* sect. *Platycarpae* (*M. ruthenica*, *M. archiducis-nicolai*, *M. platycarpos*, and *M. edgeworthii*); Clade 2 contained four species *Medicago lupulina* (sect. *Lupularia*), *Medicago minima*, *M. polymorpha*, and *M. truncatula* (sect. *Sphaerocarpos*), and *M. sativa* complex comprising three species, *M. sativa*, *Medicago falcata*, and *Medicago × varia* (sect. *Medicago*); Clade 3 contained four “medicagoid” species (*T. cancellata*, *T. arcuata*, *M. monantha*, and *M. orthoceras*; sect. *Buceras*); while other three *Trigonella* species (*T. emodi*, *T. fimbriata*, and *T. cachemiriana*; sect. *Ellipticae*) were grouped as Clade 5. Clade 4 included all the four *Melilotus* species. Clades 4 and 5 were basal to all other groups and the four “medicagoid” species (Clade 3) were reciprocally monophyletic to all *Medicago* species (Clades 1 and 2). All the five clades were strongly supported in both ML and BI analyses, except for Clade 2, which had a bootstrap value of 78 (Figure 2).

In contrast, phylogenetic relationships among and within the Clades 1–3 in the nrDNA tree were incongruent with those in the cpDNA tree (Figure 2). For example, *M. edgeworthii* was grouped with the four “medicagoid” species, which was nested within *Medicago* clade. Clade 2 in the cpDNA tree was split into two clades in the nrDNAS tree both with high support. Clade 1 was moved to a basal position in the nrDNA tree. In general, phylogenetic relationships among species within each clade received higher support in the nrDNA tree compared to those in the cpDNA tree. Moreover, the three species of *M. sativa* complex or the two *Trigonella* species (*T. emodi* and *T. fimbriata*) were always grouped together and could not be separated from each other in both phylogenetic trees.

### Morphological Clustering Based on the Morphological Traits

**FIGURE 3:**
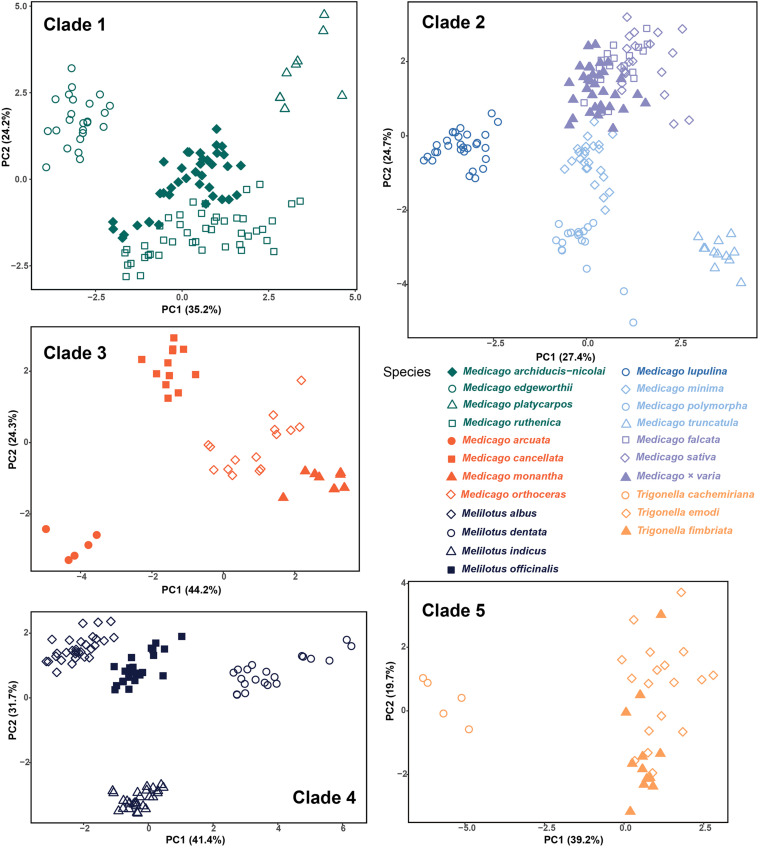
Morphological clustering within each of the five clades inferred from cpDNA phylogenetic tree based on the principal component analysis. Colors are the same as in Figure 2.

For statistical analysis, we used 12–20 morphological characters that were obviously variable between species within each clade. The detailed information of characters used for each clade are listed in Supplementary Table 4. Cumulative values of the first two principal components for the five clades ranged from 51.2 to 73.1% (Figure 3). Morphological clusters identified by PCA were generally formed by samples from the same species, and the distances among clusters were consistent with the delimitation indicated by the phylogenetic trees. In Clade 1, the four species were mostly separate with some overlap between the two sister species, *M. ruthenica* and *M. archiducis-nicolai*. In Clade 2, the three species of *M. sativa* complex were clustered together as indicated by the phylogenetic tree, whereas other species formed distinct clusters. Clade 4 had similar clustering pattern as Clade 1, where overlap was present between *Melilotus indicus* and *Melilotus officinalis*, which was not highly supported as two distinct species although samples from each species seemed to group together in the phylogenetic trees. The four “medicagoid” species (Clade 3) formed generally distinct clusters, while the two *Trigonella* species (*T. emodi* and *T. fimbriata*) could not be distinguished based on morphological characters as shown for Clade 5. Finally, the four “medicagoid” species were morphologically closer to *M. edgeworthii* than the other *Trigonella* species (see Supplementary Figure 3).

## Discussion

### Discrimination Power of the Four DNA Markers

DNA barcodes are standardized fragments of DNA that were developed with the aim of distinguishing between both closely and distantly related species of different genera and families (Hebert et al., 2003; Kress et al., 2005; Cowan et al., 2006). Multiple cpDNA markers, such as the two used in this study (*trnK-matK* and *psbA-trnH*) as well as *trnL-F*, *rbcL*, and *matK* are commonly used as core barcodes for plants (Kress and Erickson, 2007; CBOL Plant Working Group et al., 2009). The nuclear ITS was also found to show high effectiveness in discriminating species boundaries on the basis of results from large comparative dataset or between closely related species (Li et al., 2011; Pang et al., 2011; Hu et al., 2015; Lu et al., 2016), and thus was strongly recommended as an additional core barcode for plants. However, we found that the discrimination power of the nuclear marker *GA3ox1* is the highest compared with that of the three core barcodes (i.e., *trnK-matK* and *psbA-trnH* and ITS; Supplementary Figure 2), as also suggested by Steele et al. (2010). Many previous studies have suggested that ITS are more effective in delimitating closely related species than the cpDNA barcodes, and ascribed the explanation to its fast rate of mutation and lineage sorting compared with the maternally inherited cpDNAs in angiosperms (Wang et al., 2011; Hu et al., 2015; Lu et al., 2016). However, among the three core barcodes, although ITS has the highest percentage of SNPs, it has similar discrimination power for closely related species compared with that of cpDNA barcode *trnK-matK*. Phylogenetic tree reconstructed based on *trnK-matK* could delimit *M. ruthenica* + *M. archiducis-nicolai* + *M. platycarpos* but not the four “medicagoid” species, while the ITS tree resolved the opposite, in which the latter could be delimited but not the former. Our results, therefore, suggest that the discrimination powers of ITS, *GA3ox1* and two barcodes of cpDNA sequences are different, as also shown in many other studies (Su et al., 2015; Lu et al., 2016). The discrimination power of previously developed core barcodes may vary greatly in different lineages, and applying new specific markers (in this case, *GA3ox1*) may be useful when the commonly used barcodes exhibit relatively low resolution in species delimitation.

### Species Delimitation and Interspecific Relationship

As we only focused on the species occurring in China, further studies involving all species from all over the world would be needed to gain a comprehensive understanding of species delimitation in these three genera. Nevertheless, using an integrative approach based on evidence from phylogenetic data and statistical analyses of morphological variation, our results suggest that 16 (15 naturally distributed and one introduced-*M. polymorpha*) out of the 21 Chinese species can be delimited with high support values in the phylogenetic trees and distinct morphological clusters (Figures 2, 3), while the remaining five species need further investigation. Although our sampling is limited to species only from China, our results still have two taxonomic implications. First, in the Flora of China, *M. falcata* is described as a wild species and widely distributed in North China, while in other studies, this species has been treated as a subspecies of *M. sativa* (Quiros and Bauchan, 1988; Small and Jomphe, 1989). The two species *M. sativa* and *M. falcata* and their hybrid *Medicago × varia* could not be distinguished based on the molecular and morphological evidence and therefore support the later treatment that they are three subspecies in the *M. sativa* complex. Second, similarly, *T. emodi* Bentham and *T. fimbriata* Royle ex Bentham can’t be separated in neither phylogenetic analyses nor morphological clusters. The two species co-occur in southwestern Tibet of China and adjacent countries, such as India, Pakistan, and Nepal, and are distinguished mainly by the number of sawtooth at leaflet margin (Xu et al., 2010). By checking the specimens of *T. fimbriata* in KUN, we found that the species was probably established based on only few specimens due to its remote distribution. Our statistical analyses of morphological variation suggest that *T. emodi* and *T. fimbriata* cannot be distinguished from each other based on the studied morphological characters (Figure 3). These characters exhibit considerable variation between individuals even within the same population. Phylogenetic analyses also indicate that the two species form one monophyletic group. We, therefore, propose to merge the two species as one species or treat them as two subspecies.

“*Medicagoid*” species were established by Baum (1968) on the basis of similarities in flower and seed structure with those of *Medicago*, although other characters, such as the appearance of fruit, have maintained them as *Trigonella* species for a long time. Based on the tripping pollination mechanism and molecular phylogeny, Small (1987) and Bena (2001) suggested that the “*Medicagoid*” species should be transferred to the genus *Medicago*. In the Flora of China, four “*Medicagoid*” species are recorded and two of them (i.e., *T. monantha* and *T. orthoceras*) have already been revised as *Medicago* species (*M. monantha* and *M. orthoceras*). Our results indicated that the four “*Medicagoid*” species formed a monophyletic group and nested within *Medicago*. Therefore, the other two species (i.e., *T. arcuata* and *T. cancellata*) should be also transferred to *Medicago*.

Although the clades inferred in the cpDNA and nrDNA phylogenetic trees all received strong statistical supports, the relationships among them are incongruent in the two trees. Except for the two basal clades 4 and 5 (i.e., *Trigonella/Melilotus* clade), deep relationships among the early diverging lineages within *Medicago* (including the four “*Medicagoid*” species, see section “Discussion” above) recovered in the two trees are inconsistent. Such discordance of gene trees derived from nuclear and cytoplasmic markers has been found in numerous studies and usually explained by two non-exclusive factors, i.e., hybridization and introgression, and/or incomplete lineage sorting (Degnan and Rosenberg, 2009; Lemmon and Lemmon, 2013; Zwickl et al., 2014; Alexander et al., 2015; Ren et al., 2015). Hybridization/introgression often occur among closely related species where reproductive isolation is incomplete, leading to fast introgression of maternally inherited cpDNA fragments and the concerted evolution of the nuclear genes, which could distort phylogenetic relationships of closely related species (Mallet, 2007; Abbott et al., 2010). Hybridization has been indicated to be common and ongoing among lineages since the origin of *Medicago* (Maureira-Butler and Pfeil, 2008), although, it is currently unclear to what degree species in *Medicago* hybridize with each other. The incongruent placement of *M. edgeworthii* between cpDNA and nrDNA gene trees seems to be explained by hybridization. *M. edgeworthii* and the four “*Medicagoid*” species are currently co-distributed in central Asia. It is likely that these species may contact and hybridize with each other. Furthermore, hybridization/introgression scenario seems to be supported by the incongruent relationships of the three closely related species (i.e., *M. ruthenica*, *M. archiducis-nicolai*, *M. platycarpos*). However, incomplete lineage sorting that causes the retention of ancestral polymorphism in different species and/or sampling error cannot be completely excluded because of the relatively small sampling size. We, therefore, recognized that population genomic level data and more species sampling would be necessary to clearly discriminate among these possible scenarios.

## Data Availability Statement

The data presented in the study are deposited in the NCBI repository (https://www.ncbi.nlm.nih.gov/), accession numbers: MW241635–MW242342.

## Author Contributions

GR and JL conceived and designed the project. JC, WG, and MY collected the materials. JC and GW carried out the lab work and measured the morphological traits. JC, SW, and AL performed the analysis. JC and GR wrote the manuscript with the help from NS and JL. All authors read and approved the final version of the manuscript.

## Conflict of Interest

The authors declare that the research was conducted in the absence of any commercial or financial relationships that could be construed as a potential conflict of interest.

## Footnotes

1 http://www.iplant.cn/foc/

2 http://www.nsii.org.cn

## Supplementary Material

Supplementary Figure 1 Morphological clustering of all the 22 species based on the principal component analysis. Colors are the same as in Figure 2.

Supplementary Figure 2 Phylogenetic tree based on the analyses of each of the four DNA markers for the sampled individuals showing the phylogenetic relationships of all the 21 species occurring in China. **(A)** *GA3ox1*; **(B)** ITS; **(C)** *trnK-matK*; **(D)** *psbA-trnH*. Statistical support from maximum likelihood/Bayesian analyses are given above branches. The number in bracket indicate the number of individuals used for phylogenetic analyses.

Supplementary Figure 3 Morphological clustering of the four “*Medicagoid*” species, three *Trigonella* species and *Medicago edgeworthii* based on the principal component analysis. Colors are the same as in Figure 2.

Supplementary Table 1 Locations of populations and number of individuals from each population used for the nrDNA and cpDNA phylogenetic analyses.

Supplementary Table 2 The primer pairs used in this study.

Supplementary Table 3 GenBank accession numbers for DNA sequence data of *Medicago truncatula* and four *Trifolium* species used for phylogenetic analyses.

Supplementary Table 4 Morphological characters re-examined in Clades 1–5, respectively.

Supplementary Table 5 The distributions of SNPs identified for the haplotypes of the concatenated cpDNA and nrDNA datasets, respectively.
