# Supplementary material for: Phylogeny and Species Delimitation of Chinese Medicago (Leguminosae) and Its Relatives Based on Molecular and Morphological Evidence
Source: Front Plant Sci. 2021 Jan 13;11:619799. doi: 10.3389/fpls.2020.619799 (PMC7874099; doi:10.3389/fpls.2020.619799)
Supplement: Supplementary Figure 1 — Morphological clustering of all the 22 species based on the principal component analysis. Colors are the same as in Figure 2. [file Data_Sheet_1.docx]

## Supplementary Material

**Fig. S1.** Morphological clustering of all the 22 species based on the principal component analysis. Colors are the same as in Fig. 2.

#### **
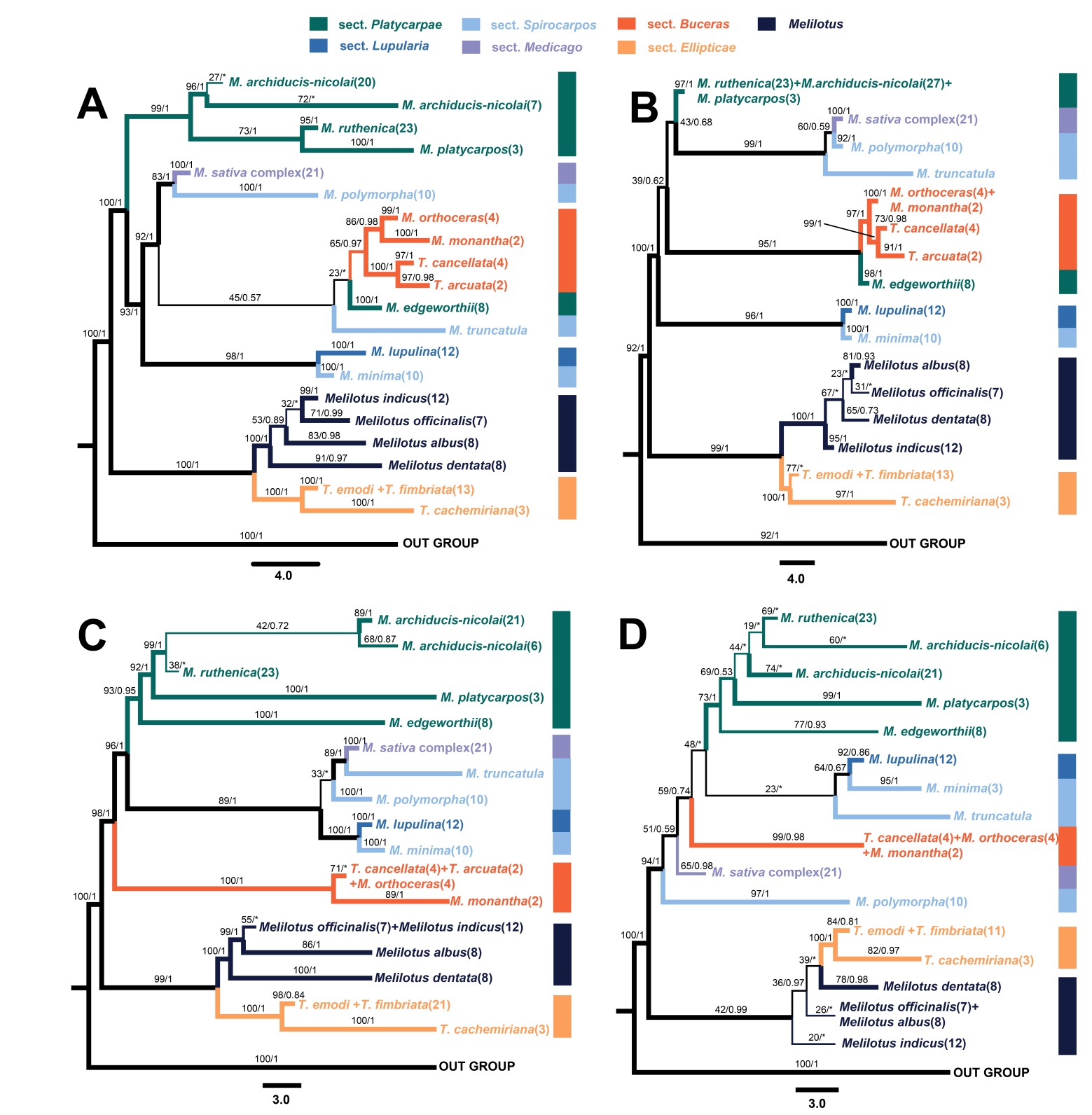
Fig. S2.** Phylogenetic tree based on the analyses of each of the four DNA markers for the sampled individuals showing the phylogenetic relationships of all the 21 species occurring in China. A, *GA3ox1*; B, ITS; C, *trnK-matK*; D, *psbA-trnH*. Statistical support from maximum likelihood/Bayesian analyses are given above branches. The number in bracket indicate the number of individuals used for phylogenetic analyses.

####
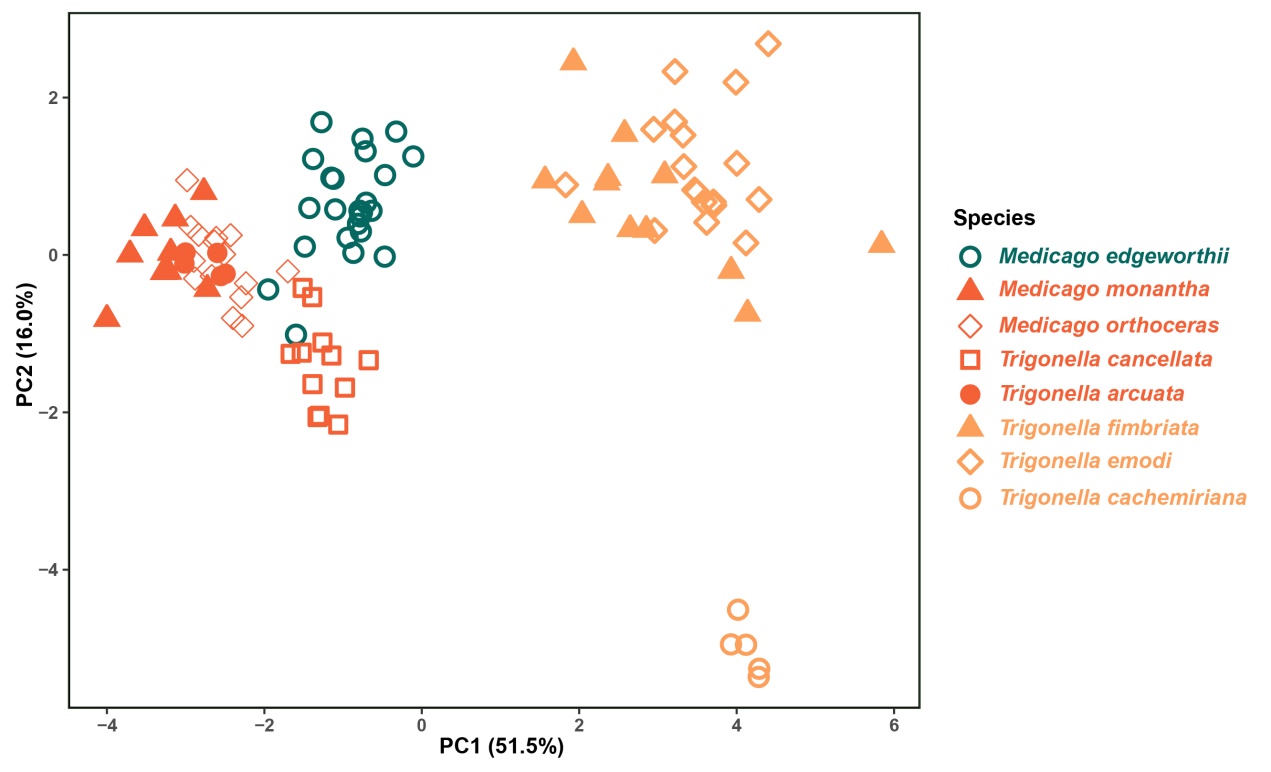


#### **Fig. S3.** Morphological clustering of the four “*Medicagoid*” species, three *Trigonella* species and *Medicago edgeworthii* based on the principal component analysis. Colors are the same as in Fig. 2.

#### **Table S1.** Locations of populations and number of individuals from each population used for the nrDNA and cpDNA phylogenetic analyses.

| population code | Location | Longitude/° | Latitude /° | Altitude /m | N1  (nrDNA) | N2  (cpDNA) | Sample ID |
| --- | --- | --- | --- | --- | --- | --- | --- |
| ***Melilotus dentata*** | | | | | | | |
| 1 | Yanggao, Shanxi | 113.877583 | 40.370711 | 1020 | H1(2) | C1(2) | S1/S2 |
| 2 | Nanniwan, Shannxi | 109.645447 | 36.330725 | 1102 | H1(1) | C1(1) | S4 |
| 3 | Zhangwu, Liaoning | 122.537882 | 42.706456 | 216 | H1(2) | C1(2) | S5/S6 |
| 4 | Yancheng, Jiangsu | 120.417661 | 33.567088 | 13 | H1(1) | C1(1) | S287 |
| 5 | Turpan, Xinjiang | 89.244129 | 43.013319 | 164 | H1(1) | C1(1) | S288 |
| 6 | Longde, Ningxia | 106.166323 | 35.569952 | 2220 | H1(1) | C1(1) | S289 |
| ***Melilotus albus*** | | | | | | | |
| 1 | Urumqi, Xinjiang | 87.580669 | 43.847976 | 813 | H4(1) | C3(1) | S35 |
| 2 | Gongliu, Xinjiang | 81.988456 | 43.580593 | 728 | H4(1) | C3(1) | S36 |
| 3 | Buerjin, Xinjiang | 87.13922 | 48.490331 | 1574 | H4(1) | C3(1) | S38 |
| 4 | Ji'nan, Shandong | 117.047396 | 36.627213 | 281 | H4(1) | C3(1) | S40 |
| 5 | Kedong, Heilongjiang | 126.261256 | 48.058965 | 335 | H4(1) | C3(1) | S41 |
| 6 | Ledu, Qinghai | 102.229008 | 36.476781 | 2055 | H4(1) | C3(1) | S42 |
| 7 | Guide, Qinghai | 101.39986 | 36.021136 | 2229 | H4(1) | C3(1) | S43 |
| 8 | Maoxian, Sichuan | 103.588955 | 31.805058 | 1933 | H4(1) | C3(1) | S44 |
| ***Melilotus officinalis*** | | | | | | | |
| 1 | Luhuo, Sichuan | 100.626805 | 31.419389 | 3220 | H2(1) | C2(1) | S8 |
| 2 | Guide, Qinghai | 101.39986 | 36.021136 | 2229 | H2(1) | C2(1) | S15 |
| 3 | Ji'nan, Shandong | 117.047396 | 36.627213 | 281 | H2(1) | C2(1) | S20 |
| 4 | Baihuashan, Beijing | 115.595568 | 39.824766 | 1815 | H2(1) | C2(1) | S21 |
| 5 | Qitai, Xinjiang | 89.758285 | 43.573598 | 2101 | H2(1) | C2(1) | S25 |
| 6 | Huocheng, Xinjiang | 81.178794 | 44.456475 | 1973 | H2(1) | C2(1) | S27 |
| 7 | Taxkorgan, Xinjiang | 75.212001 | 37.789348 | 3080 | H2(1) | C2(1) | S292 |
| ***Melilotus indicus*** | | | | | | | |
| 1 | Jiuzhaigou, Sichuan | 104.224963 | 33.208268 | 2118 | H3(3) | C2(3) | S277/S278/S279 |
| 2 | Barkam, Sichuan | 102.282663 | 31.847999 | 2743 | H3(1) | C2(1) | S10 |
| 3 | Maoxian, Sichuan | 103.588955 | 31.805058 | 1933 | H3(1) | C2(1) | S11 |
| 4 | Dacai, Qinghai | 101.48111 | 36.49305 | 2732 | H3(1) | C2(1) | S14 |
| 5 | Pianguan, Shanxi | 111.480935 | 39.46852 | 1029 | H3(1) | C2(1) | S16 |
| 6 | Tongyu, Jilin | 123.12499 | 44.795544 | 148 | H3(1) | C2(1) | S18 |
| 7 | Beian, Heilongjiang | 126.501868 | 48.289043 | 279 | H3(1) | C2(1) | S22 |
| 8 | Yongdeng, Gansu | 102.825165 | 36.607329 | 2018 | H3(3) | C2(3) | S280/S281/S282 |
| 3 | Guangzhou, Guangdong | NA | NA | NA | NA | C2(1) | S351 |
| ***Medicago sativa*** | | | | | | | |
| 1 | Buerjin, Xinjiang | 87.13922 | 48.490331 | 1574 | H20(1) | C17(1) | S159 |
| 2 | Qitai, Xinjiang | 89.758285 | 43.573598 | 2101 | H26(1) | C17(1) | S162 |
| 3 | Baihuashan, Beijing | 115.595568 | 39.824766 | 1815 | H20(1) | C17(1) | S168 |
| 4 | Zhangwu, Liaoning | 122.537882 | 42.706456 | 216 | H20(1) | C17(1) | S171 |
| 5 | Beian, Heilongjiang | 126.459589 | 48.262534 | 255 | H20(1) | C17(1) | S174 |
| 6 | Dong Ujimqin Banner, Inner Mongolia | 117.007244 | 45.545442 | 871 | H20(1) | C17(1) | S177 |
| 7 | Dacai, Qinghai | 101.48111 | 36.49305 | 2732 | H26(1) | C17(1) | S183 |
| 8 | Dingqing, Tibet | 95.827853 | 31.273609 | 3551 | H26(1) | C17(1) | S191 |
| 9 | Jilong, Tibet | 85.295858 | 28.8477 | 4098 | H26(1) | C19(1) | S374 |
| 10 | Diebu, Gansu | 103.874624 | 34.219102 | 2784 | H20(1) | C17(1) | S379 |
| ***Medicago falcata*** | | | | | | | |
| 1 | Urumqi, Xinjiang | 87.478553 | 43.443868 | 1783 | H18(1) | C15(1) | S128 |
| 2 | Urumqi, Xinjiang | 87.580669 | 43.847976 | 813 | H19(1) | C15(1) | S134 |
| 3 | Gongliu, Xinjiang | 82.688942 | 43.272979 | 1101 | H20(1) | C15(1) | S136 |
| 4 | Huocheng, Xinjiang | 81.178794 | 44.456475 | 1973 | H21(1) | C15(1) | S138 |
| 5 | Buerjin, Xinjiang | 87.13922 | 48.490331 | 1574 | H22(1) | C15(1) | S140 |
| 6 | Zhaosu, Xinjiang | 80.282437 | 42.690842 | 1842 | H25(1) | C16(1) | S365 |
| ***Medicago*** ***× varia*** | | | | | | | |
| 1 | Urumqi, Xinjiang | 87.478553 | 43.443868 | 1783 | H20(1) | C18(1) | S142 |
| 2 | Urumqi, Xinjiang | 87.580669 | 43.847976 | 813 | H20(1) | C17(1) | S146 |
| 3 | Gongliu, Xinjiang | 82.688942 | 43.272979 | 1101 | H19(1) | C16(1) | S152 |
| 4 | Huocheng, Xinjiang | 81.178794 | 44.456475 | 1973 | H23(1) | C17(1) | S155 |
| 5 | Heshigten Banner, Inner mongolia | 117.527578 | 43.260957 | 1059 | H24(1) | C17(1) | S158 |
| ***Medicago edgeworthii*** | | | | | | | |
| 1 | Dege, Sichuan | 98.641146 | 31.589428 | 3025 | H13(1) | C9(1) | S120 |
| 2 | Chaya, Tibet | 97.343565 | 30.722507 | 3178 | H14(1) | C9(1) | S123 |
| 3 | Dingqing, Tibet | 95.827853 | 31.273609 | 3551 | H15(1) | C9(1) | S124 |
| 4 | Nangqian, Qinghai | 96.499564 | 32.182763 | 3583 | H15(1) | C9(1) | S126 |
| 5 | Yushu, Qinghai | 96.977863 | 33.005775 | 3743 | H16(1) | C9(1) | S127 |
| 6 | Nielamu, Tibet | 85.970857 | 28.165494 | 3763 | H17(2) | C9(2) | S359/S360 |
| 7 | Jilong, Tibet | 85.302936 | 28.86181 | 4138 | H16(1) | C10(1) | S361 |
| ***Medicago platycarpos*** | | | | | | | |
| 1 | Fuhai, Xinjiang | 88.953509 | 47.868789 | 1343 | H32(3) | C20(3) | S249/S250/S251 |
| ***Medicago lupulina*** | | | | | | | |
| 1 | Gongliu, Xinjiang | 81.988456 | 43.580593 | 728 | H5(1) | C4(1) | S49 |
| 2 | Huocheng, Xinjiang | 81.178794 | 44.456475 | 1973 | H5(1) | C4(1) | S51 |
| 3 | Shennongjia, Hubei | 110.335683 | 31.539967 | 1729 | H5(1) | C4(1) | S52 |
| 4 | Kedong, Heilongjiang | 126.261256 | 48.058965 | 335 | H5(1) | C4(1) | S55 |
| 5 | Heshigten Banner, Inner mongolia | 117.527578 | 43.260957 | 1059 | H5(1) | C4(1) | S56 |
| 6 | Nanniwan, Shannxi | 109.645447 | 36.330725 | 1102 | H5(1) | C4(1) | S58 |
| 7 | Guide, Qinghai | 101.39986 | 36.021136 | 2229 | H5(1) | C4(1) | S61 |
| 8 | Aba, Sichuan | 101.67439 | 32.921745 | 3303 | H5(1) | C4(1) | S62 |
| 9 | Lixian, Sichuan | 102.77004 | 31.610118 | 2941 | H5(1) | C4(1) | S64 |
| 10 | Dege, Sichuan | 98.641146 | 31.589428 | 3025 | H5(1) | C4(1) | S67 |
| 11 | Jiangda, Tibet | 98.386471 | 31.592717 | 3207 | H5(1) | C4(1) | S68 |
| 12 | Yixian, Anhui | 117.95765 | 29.893113 | 197 | H5(1) | C4(1) | S316 |
| ***Medicago archiducis-nicolai*** | | | | | | | |
| 1 | Yongdeng, Gansu | 103.050535 | 36.841215 | 2605 | H12(1) | C6(1) | S204 |
| 2 | Ping'an, Qinghai | 101.9111 | 36.33666 | 2736 | H12(1) | C6(1) | S205 |
| 3 | Ganhetan, Qinghai | 101.55166 | 36.5444 | 2736 | H12(1) | C6(1) | S206 |
| 4 | Dacai, Qinghai | 101.48111 | 36.49305 | 2732 | H12(1) | C6(1) | S207 |
| 5 | Huangzhong, Qinghai | 101.560725 | 36.489725 | 2752 | H12(1) | C6(1) | S208 |
| 6 | Guide, Qinghai | 101.243917 | 35.781543 | 3329 | H12(1) | C6(1) | S209 |
| 7 | Guinan, Qinghai | 100.849111 | 35.510972 | 3312 | H12(1) | C6(1) | S210 |
| 8 | Tongde, Qinghai | 100.64777 | 35.262777 | 3290 | H12(1) | C6(1) | S211 |
| 9 | Banma, Qinghai | 100.772583 | 33.275138 | 3913 | H8(1) | C7(1) | S212 |
| 10 | Aba, Sichuan | 101.845574 | 32.915826 | 3592 | H9(1) | C7(1) | S213 |
| 11 | Barkam, Sichuan | 102.282663 | 31.847999 | 2743 | H10(1) | C7(1) | S214 |
| 12 | Seda, Sichuan | 100.738655 | 31.831923 | 3608 | H10(1) | C7(1) | S215 |
| 13 | Ganzi, Sichuan | 100.217771 | 31.617349 | 3929 | H9(1) | C7(1) | S216 |
| 14 | Dege, Sichuan | 99.146167 | 31.893572 | 4011 | H9(1) | C7(1) | S217 |
| 15 | Dege, Sichuan | 98.641146 | 31.589428 | 3025 | H12(1) | C8(1) | S218 |
| 16 | Jiangda, Tibet | 98.386471 | 31.592717 | 3207 | H12(1) | C6(1) | S219 |
| 17 | Changdu, Tibet | 97.205873 | 31.405784 | 3309 | H12(1) | C6(1) | S220 |
| 18 | Chaya, Tibet | 97.343565 | 30.722507 | 3178 | H8(1) | C6(1) | S221 |
| 19 | Dingqing, Tibet | 95.827853 | 31.273609 | 3551 | H12(1) | C6(1) | S222 |
| 20 | Leiwuqi, Tibet | 96.594833 | 31.253838 | 3779 | H11(1) | C6(1) | S223 |
| 21 | Nangqian, Qinghai | 96.499564 | 32.182763 | 3583 | H9(1) | C6(1) | S224 |
| 22 | Yushu, Qinghai | 97.049855 | 32.852749 | 3804 | H12(1) | C6(1) | S225 |
| 23 | Daotanghe, Qinghai | 100.71475 | 36.527305 | 3235 | H12(1) | C6(1) | S226 |
| 24 | Tiebujia, Qinghai | 99.544 | 37.066194 | 3239 | H12(1) | C6(1) | S227 |
| 25 | Menyuan, Qinghai | 101.404591 | 37.575089 | 3094 | H12(1) | C6(1) | S228 |
| 26 | Longde, Ningxia | 106.166323 | 35.569952 | 2220 | H12(1) | C6(1) | S353 |
| 27 | Tianzhu, Gansu | 102.783647 | 37.194766 | 2865 | H12(1) | C6(1) | S200 |
| ***Medicago ruthenica*** | | | | | | | |
| 1 | Zoucheng, Shandong | 117.325212 | 35.387084 | 616 | H6(1) | C5(1) | S70 |
| 2 | Baihuashan, Beijing | 115.595568 | 39.824766 | 1815 | H7(1) | C5(1) | S71 |
| 3 | Zhangwu, Liaoning | 122.537882 | 42.706456 | 216 | H7(1) | C5(1) | S72 |
| 4 | Daqing, Heilongjiang | 124.510333 | 46.822736 | 139 | H6(1) | C5(1) | S73 |
| 5 | Kedong, Heilongjiang | 126.261256 | 48.058965 | 335 | H6(1) | C5(1) | S74 |
| 6 | Beian, Heilongjiang | 126.501868 | 48.289043 | 279 | H6(1) | C5(1) | S75 |
| 7 | Manzhouli, Inner mongolia | 117.450015 | 49.559565 | 708 | H7(1) | C5(1) | S76 |
| 8 | Zhenlai, Jilin | 123.008223 | 46.168251 | 156 | H6(1) | C5(1) | S77 |
| 9 | Tongyu, Jilin | 123.12499 | 44.795544 | 148 | H6(1) | C5(1) | S78 |
| 10 | Horqin Right Wing Middle Banner, Inner mongolia | 121.480402 | 45.112569 | 299 | H6(1) | C5(1) | S79 |
| 11 | Huolinguole, Inner mongolia | 119.639336 | 45.578869 | 872 | H6(1) | C5(1) | S80 |
| 12 | Dong Ujimqin Banner, Inner Mongolia | 117.007244 | 45.545442 | 871 | H7(1) | C5(1) | S81 |
| 13 | Balin left Banner, Inner Mongolia | 119.454623 | 43.990489 | 509 | H7(1) | C5(1) | S82 |
| 14 | Ongniud Banner, Inner Mongolia | 118.999567 | 42.976253 | 652 | H7(1) | C5(1) | S83 |
| 15 | Chifeng, Inner Mongolia | 119.023505 | 42.290726 | 597 | H7(1) | C5(1) | S84 |
| 16 | Heshigten Banner, Inner mongolia | 117.527578 | 43.260957 | 1059 | H7(1) | C5(1) | S85 |
| 17 | Wulanchabu, Inner mongolia | 113.21785 | 40.902361 | 1285 | H7(1) | C5(1) | S86 |
| 18 | Yanggao, Shanxi | 113.738715 | 40.232051 | 1098 | H7(1) | C5(1) | S87 |
| 19 | Pianguan, Shanxi | 111.480935 | 39.46852 | 1029 | H7(1) | C5(1) | S88 |
| 20 | Huhehot, Inner mongolia | 111.71089 | 40.906289 | 1137 | H6(1) | C5(1) | S89 |
| 21 | Nanniwan, Shannxi | 109.645447 | 36.330725 | 1102 | H6(1) | C5(1) | S90 |
| 22 | Ledu, Qinghai | 102.229008 | 36.476781 | 2055 | H6(1) | C5(1) | S91 |
| 23 | Maoxian, Sichuan | 103.588955 | 31.805058 | 1933 | H6(1) | C5(1) | S92 |
| ***Medicago polymorpha*** | | | | | | | |
| 1 | Cangxi, Sichuan | 106.005842 | 31.94636 | 358 | H34(1) | C21(1) | S254 |
| 2 | Zigui, Hubei | 110.745275 | 31.029707 | 211 | H34(1) | C21(1) | S255 |
| 3 | Tongcheng, Anhui | 116.929191 | 31.045463 | 51 | H34(1) | C21(1) | S256 |
| 4 | Yixian, Anhui | 117.95765 | 29.893113 | 197 | H34(1) | C21(1) | S258 |
| 5 | Jintang, Zhejiang | 121.87626 | 30.035685 | 20 | H34(1) | C21(1) | S259 |
| 6 | Lin'an, Zhejiang | 119.772282 | 30.189571 | 43 | H34(1) | C21(1) | S260 |
| 7 | Maanshan, Anhui | 118.255513 | 31.772749 | 27 | H34(1) | C21(1) | S261 |
| 8 | Yancheng, Jiangsu | 120.417661 | 33.567088 | 13 | H34(2) | C21(2) | S262/S263 |
| 9 | Chenggu, Shannxi | 107.346315 | 33.180571 | 473 | H34(1) | C21(1) | S264 |
| ***Medicago minima*** | | | | | | | |
| 1 | Wudu, Gansu | 104.949069 | 33.353333 | 1246 | H36(1) | NA | S265 |
| 2 | Zigui, Hubei | 110.745275 | 31.029707 | 211 | H36(1) | NA | S266 |
| 3 | Jixi, Anhui | 118.609468 | 30.104193 | 189 | H36(1) | NA | S267 |
| 4 | Lianyungang, Jiangsu | 118.430968 | 34.440335 | 78 | H36(1) | NA | S268 |
| 5 | Fengqiu, Henan | 114.734106 | 34.995611 | 62 | H36(1) | NA | S269 |
| 6 | Zhengzhou, Henan | 113.663446 | 34.911051 | 68 | H36(1) | NA | S270 |
| 7 | Fengxiang, Shannxi | 107.388779 | 34.524257 | 804 | H36(1) | C22(1) | S272 |
| 8 | Chenggu, Shannxi | 107.346315 | 33.180571 | 474 | H36(3) | C22(2) | S273/S275/S276 |
| ***Trigonella cancellata*** | | | | | | | |
| 1 | Miaoergou, Xinjiang | 87.011375 | 43.5266 | 1505 | H27(1) | C11(1) | S194 |
| 2 | Zhaosu, Xinjiang | 80.282437 | 42.690842 | 1842 | H27(1) | C11(1) | S229 |
| 3 | Tekesi, Xinjiang | 81.672317 | 43.159855 | 1276 | H27(1) | C11(1) | S230 |
| 4 | Xinyuan, Xinjiang | 83.837813 | 43.382349 | 1127 | H27(1) | C11(1) | S231 |
| ***Medicago monantha*** | | | | | | | |
| 1 | Gongliu, Xinjiang | 82.688942 | 43.272979 | 1101 | H29(1) | C12(2) | S197/S198 |
| ***Medicago orthoceras*** | | | | | | | |
| 1 | Zhaosu, Xinjiang | 80.282437 | 42.690842 | 1842 | H30(1) | C13(1) | S237 |
| 2 | Tekesi, Xinjiang | 81.672317 | 43.159855 | 1276 | H30(1) | C13(1) | S238 |
| 3 | Xinyuan, Xinjiang | 83.837813 | 43.382349 | 1127 | H31(1) | C13(1) | S240 |
| 4 | Gongliu, Xinjiang | 82.688942 | 43.272979 | 1101 | H30(1) | C13(1) | S199 |
| ***T******rigonella arcuata*** | | | | | | | |
| 1 | Huocheng, Xinjiang | 80.919706 | 44.040862 | 596 | H28(2) | NA | S352/S353 |
| ***Trigonella cachemiriana*** | | | | | | | |
| 1 | Taxkorgan, Xinjiang | 75.212001 | 37.789348 | 3080 | H33(3) | C23(3) | S243/S252/S253 |
| ***Trigonella emodi*** | | | | | | | |
| 1 | Nielamu, Tibet | 85.970857 | 28.165494 | 3763 | H37(1)/H38(1)/ H39(1) | C24(3) | S246/S385/S386/S347 |
| 2 | Jilong, Tibet | 85.302936 | 28.86181 | 4138 | H37(2) | C24(2) | S247/S248/S387/S348 |
| 3 | Cuona, Tibet | NA | NA | NA | H37(1) | NA | S345/S346 |
| 4 | Nepal | NA | NA | NA | H37(1) | NA | S349 |
| 5 | Longzi, Tibet | NA | NA | NA | NA | NA | S350 |
| ***Trigonella fimbriata*** | | | | | | | |
| 1 | Nielamu, Tibet | 85.970857 | 28.165494 | 3763 | H37(1)/H38(1) | C24(3) | S244/S381/S382/S342  /S343/S344 |
| 2 | Jilong, Tibet | 85.302936 | 28.86181 | 4138 | H37(3) | C24(3) | S245/S383/S384 |
| Total |  |  |  |  | 176 | 167 | 186 |

#### **Table S2.** The primer pairs used in this study.

| Primer name | | Primer sequence | Tm |
| --- | --- | --- | --- |
| *trnK*-*matK* | trnK 1L | CTCAATGGTAGAGTACTCG | 50 ℃ |
|  | trnK 708R | TCAAATGATACATAGTGCGATAC |  |
|  | trnK 685F | GTATCGCACTATGTATCATTTGA | 50 ℃ |
|  | matK 1932R | CAGACCGGCTTACTAATGGG |  |
|  | matK 4L | CTTCGCTACTGGGTGAAAGATG | 59 ℃ |
|  | trnK 2R | AACTAGTCGGATGGAGTAG |  |
| *psbA-trnH* | psbA | GTTATGCATGAACGTAATGCTC | 54 ℃ |
|  | trnH | CGCGCATGGTGGATTCACAAATC |  |
| *GA3ox1* | GA3ox 3F | CTCCTCCTTCTTCCCCAAACTCA | 58 ℃ |
|  | GA3ox 20R | GTGCCAAGGTACTCATTCC |  |
| ITS | ITS F | TCCGTAGGTGAACCTGCGG | 58 ℃ |
|  | ITS R | TTCCTCGCTTATTGATATGTTAAACTC |  |

| Species | *trnK-matK* | *psbA-trnH* | *GA3ox1* | ITS |
| --- | --- | --- | --- | --- |
| *Medicago truncatula* | NC_003119.6 | JQ734438 | AC208096 | Z92923 |
| *Trifolium albopurpureum* Torr. & A. Gray | AF522116 | NA | HM211171 | AF053143 |
| *Trifolium incarnatum* L. | AF522126 | NA | HM211172 | AF053160 |
| *Trifolium semipilosum* Fresen. | AF522132 | NA | HM211174 | AF053175 |
| *Trifolium subterraneum* L. | AF522135 | NA | HM211175 | AF053177 |

**Table S3.** GenBank accession numbers for DNA sequence data of *Medicago truncatula* and four *Trifolium* species used for phylogenetic analyses.
